# Comparison of Immunogen Designs That Optimize Peptide Coverage: Reply to Fischer et al

**DOI:** 10.1371/journal.pcbi.0040025

**Published:** 2008-01-25

**Authors:** David C Nickle, Nebojsa Jojic, David Heckerman, Vladimir Jojic, Darko Kirovski, Morgane Rolland, Sergei Kosakovsky Pond, James I Mullins

In our paper “Coping with Viral Diversity in HIV Vaccine Design” [1], we presented several approaches to incorporate viral variability within vaccine immunogens, including judicious choice of natural strains. Most of our approaches included at least one collinear gene length corresponding to the Center-of-Tree (COT) sequence, which has near-optimal peptide coverage for a single gene. Inclusion of a COT sequence and optimizing the rest of the immunogen for coverage, as suggested in [2], yielded a construct (COT+) with the greatest coverage of peptide diversity, minimally sacrificing peptide coverage in comparison with unconstrained diversity optimization. Fischer et al. [3] introduced mosaics—a different approach to increasing coverage while maintaining collinearity using an optimization algorithm based on simulated recombination.

**Figure 1:**
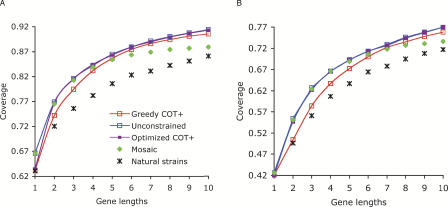
Comparison of Peptide Coverage Scores Achievable with Different Immunogen Formats and Algorithms In (A) and (B), we show the coverage for Gag and Nef, respectively, as a function of immunogen gene length for different immunogen formats. Natural strains: a cocktail of natural HIV sequences optimized for breadth of diversity by Gibbs sampling. Mosaics: a cocktail of constructs collinear with natural strains optimized by the code developed by Fischer et al. [3]. Greedy COT+: COT+ sequences were optimized by a simple greedy algorithm described by Jojic et al. [2,13] and Nickle et al. [1]. Optimized COT+: a stochastic combinatorial optimization of COT+ using the algorithm of Kirovski et al. [5], run on a 300 node Windows HPC cluster for one day. Unconstrained: a sequence optimized for coverage with the Kirovski algorithm without regard to gene collinearity. To emphasize that some of the constructs can be optimized at fractional lengths, those results are shown by lines (since points on the line are achievable), while the constructs consisting of an integer number of strains are shown as dots only.

In their response to Nickle et al. [1], Fischer et al. [4] suggest that maintaining full collinearity of viral gene sequences with native viral proteins is the only tractable approach to producing immunogens inclusive of viral variability. This claim was based on the observation that mosaics had slightly higher coverage than COT+ at 3× and 4× strain lengths, despite the fact that all mosaic components are constrained to be collinear with the full gene. However, as we pointed out, a variety of optimization algorithms can be used to perform coverage optimization, with computationally intensive approaches typically yielding better results. Figure 1 compares the coverage of mosaics with COT+ constructs produced by two optimization algorithms—the simple greedy extension described in Nickle et al. [1], which can be implemented in hours and run in seconds on any modern personal computer, and the more complex combinatiorial optimization approach of [5] run for one day on a cluster of 300 PCs. We also include the coverage of a construct optimized without any collinearity constraints, derived using the Kirovski et al. [5] algorithm. The coverage of COT+ created by combinatorial optimization is greater than that of mosaics, especially at larger lengths where even the simple greedy algorithm surpasses the mosaic coverage. Furthermore, the optimized COT+ coverage is almost identical to the coverage of constructs optimized with no collinearity constraints, indicating that the price for imposing a constraint on the immunogen to include a single virus-like strain is small.

Fischer and colleagues also argued that COT+ creates unnatural peptide sequences by concatenation. However, similar concatenation of their mosaics would have produced about 18 unnatural 9-mer peptides. Furthermore, the COT+ approach can be tuned to both penalize the introduction of unnatural peptides on concatenation, and to define the number of segments to be separately expressed, and thus reduce the requirement for concatenation.

Several additional inferences were made in the response by Fischer et al. that should be commented upon. First, COT+ may, of course, be optimized for arbitrary HIV clades or combinations of clades, but the publication of our paper in *PLoS Computational Biology* reflects our focus on approaches to immunogen design rather than on the production of an exhaustive series of constructs. Also, just as in the mosaic approach, COT+ can be optimized to exclude rare variants (referred to as smoothing in our paper).

Fischer et al. also discussed disappointing unpublished findings on the immunogenicity induced against Nef by a construct obtained by fusing a full-length Gag gene and the central portion of the Nef gene. However, these results can only be fairly assessed in light of what would be expected for the full-length Nef protein, and in the case of cellular immune responses, in the context of the same MHC specificities. However, these controls were not provided. We certainly agree that there are substantial challenges to the establishment of a multivalent CD8 response, yet multiple strategies have been and are being devised to overcome this important problem. For example, different groups have shown that CD8+ T cell responses can be successfully elicited against CD8+ T cell epitope strings when they are separated by short linker sequences and not in the context of the native protein, implying that they can be processed and presented in vivo [6–11].

Finally, despite 25 years of AIDS research and intensive yet uniformly failed efforts to develop an AIDS vaccine, the scientific community is poorly positioned to determine which, if any, approach to vaccine immunogen design will prove successful. Thus, arguing over methodologies developed with the same goal of incorporating variability has little significance as long as we do not know whether maximizing variability or inclusion of the entire full-length viral proteins are valid strategies. It may very well be that removing certain epitopes could be a more judicious approach than an overall epitope maximization strategy [12]. Indeed, the flexibility afforded by the COT+ approach, which is not limited to full-length proteins, may well prove superior to immunization with full-length viral protein immunogens.
