# Correction: Comparison of Immunogen Designs That Optimize Peptide Coverage: Reply to Fischer et al

**DOI:** 10.1371/annotation/da2fae86-8f92-4d32-aefe-a30a1de7a8f8

**Published:** 2008-03-21

**Authors:** David C Nickle, Nebojsa Jojic, David Heckerman, Vladimir Jojic, Darko Kirovski, Morgane Rolland, Sergei Kosakovsky Pond, James I Mullins

Under Funding, it was incorrectly stated that the authors had received no specific funding for their article. Funding was provided through NIHP30 AI27757 (University of Washington Center for AIDS Research) and NIH P01AI57005.

